# An Insight of RuBisCO Evolution through a Multilevel Approach

**DOI:** 10.3390/biom11121761

**Published:** 2021-11-25

**Authors:** Vladimir Camel, Gaston Zolla

**Affiliations:** 1 Grupo de Investigacion en Mutaciones & Biotecnología Vegetal, Facultad de Agronomía, Universidad Nacional Agraria La Molina, Av. La Molina s/n., Lima 12175, Peru; gzolla@lamolina.edu.pe; 2 Doctoral Program in Biological Sciences and Engineering, National Agrarian University La Molina, Av. La Molina s/n., Lima 12175, Peru; 3 Programa de Fisiología Vegetal y Cambio Climático, Asociación ANDINUS, Calle Miguel Grau 370, Sicaya, Huancayo, Junín 12500, Peru; 4 Grupo de Investigacion de Fisiología Vegetal, Facultad de Ciencias, Universidad Nacional Agraria La Molina, Av. La Molina s/n., Lima 12175, Peru

**Keywords:** Bio3D, structural dynamics, structural flexibility, cross-correlation dynamics

## Abstract

RuBisCO is the most abundant enzyme on earth; it regulates the organic carbon cycle in the biosphere. Studying its structural evolution will help to develop new strategies of genetic improvement in order to increase food production and mitigate CO_2_ emissions. In the present work, we evaluate how the evolution of sequence and structure among isoforms I, II and III of RuBisCO defines their intrinsic flexibility and residue-residue interactions. To do this, we used a multilevel approach based on phylogenetic inferences, multiple sequence alignment, normal mode analysis, and molecular dynamics. Our results show that the three isoforms exhibit greater fluctuation in the loop between αB and βC, and also present a positive correlation with loop 6, an important region for enzymatic activity because it regulates RuBisCO conformational states. Likewise, an increase in the flexibility of the loop structure between αB and βC, as well as Lys330 (form II) and Lys322 (form III) of loop 6, is important to increase photosynthetic efficiency. Thus, the cross-correlation dynamics analysis showed changes in the direction of movement of the secondary structures in the three isoforms. Finally, key amino acid residues related to the flexibility of the RuBisCO structure were indicated, providing important information for its enzymatic engineering.

## 1. Introduction

RuBisCO (ribulose-1,5-bisphosphate carboxylase oxygenase) is the most abundant enzyme in nature and plays essential functions in the entry of carbon into the biosphere and in photorespiration processes [1]. It is found in most autotrophic organisms such as bacteria, archaea and eukarya (algae, higher plants) [2]. Evolutionary studies in RuBisCO have allowed its classification into four isoforms (I, II, III and IV) [3,4]. Isoform I is the predominant enzyme in nature and is found in cyanobacteria, green algae and in higher and lower plants. It is a holoenzyme consisting of eight large (RbcL) and eight small (RbcS) subunits [5]. The isoform II enzyme is present in bacteria and is composed only of large-type subunit multimers [(L2)x], and appears to be less efficient in cleaving CO_2_ and O_2_ [4,6]. Isoform II has a distinct physiological role, and it is used primarily to allow the Calvin–Benson–Bassham pathway to balance the cell redox potential [7,8]. Isoform III is found in archaeas and consists of a toroid-shaped pentagonal decamer composed of L subunits [9]. In addition, the enzyme shows extreme thermostability with high carboxylase activity at high temperatures [10,11] and exceeds the RuBisCO activity of spinach by 20 times, but it is not efficient at room temperature [12]. Moreover, it is not affected by the presence of oxygen [9,13,14]. Isoform IV includes proteins similar to RuBisCO (RLP) but does not use CO_2_ as the main source of carbon [15]. Despite the variability of the amino acid sequences within the different RuBisCO isoforms [5,16], the key residues of the active site, catalytic chemistry and activation processes are conserved, and this supports the concept that there is a conserved set of residues that are critical for folding and maintaining the general structure of the enzyme [15,17]. However, it is possible that proteins within the same isoform may have different enzymatic and kinetic properties. For example, phylogenetic studies show that the sequence of RbcL from *Arabidopsis thaliana* (5IU0) is different from *Oryza sativa* (1WDD) despite exhibiting high structural similarity. On the other hand, the amino acid sequences of isoform III in *Methanococcoides burtonii* (5MAC) is closer to isoform II. Likewise, the RuBisCO of *Nostoc* sp. (6KKM) and *Synechococcus elongatus* (6SMH) is isoform I, but their sequences are similar to isoform III. Due to these differences, it is necessary to understand the relationship between the structure and function of the RuBisCO enzyme in order to understand the role of the residues directly involved in catalysis. Furthermore, Nishitani et al. [10] showed that mutations (SP5-V330T) in the RbcL 3A12^WT^ protein of *Thermococcus kodakarensis* increased the flexibility of the α-helix 6 and loop 6 regions, being important to increase the photosynthetic efficiency of the enzyme at room temperature. Likewise, the closure of the active site implies movements of loop 6 and flexible elements of the N-terminal domain of the adjacent subunit in the dimer [18]. Currently, the two states, open and closed, of the RuBisCO enzyme are quite well-defined structurally, but the details of the closing mechanism are still unknown [19]. Therefore, it is necessary to study the influence between the structure, the amino acid composition and the flexibility of the RuBisCO structures.

On the other hand, RuBisCO is a widely studied enzyme. Consequently, the PDB (Protein Data Bank) repository has several RbcL structures [20], which are useful to understand the evolution of the different RuBisCO isoforms. In this sense, the Bio3D [21,22] and Prody packages emerged as computational tools that help to better understand the relationship between the structure, dynamics and function of sets of evolutionarily related proteins [23,24].

Consequently, in the present work, we evaluate how the evolution of sequence and structure among isoforms I, II and III of RuBisCO defines their intrinsic flexibility and residue-residue interaction.

## 2. Materials and Methods

### 2.1. Classification of RuBisCO Isoforms

RuBisCO protein codes (1RLC, 4RUB, 4HHH, 1GK8, 4LF1 and 3A12) were used to search for homologous structures using BLAST [25]; then, the different RuBisCO structures determined by crystallography were downloaded from RCSB PDB [20] using the Bio3D package [21,22]. A sequence identity threshold of 70% was used according to Kalenkiewicz et al. [26] to isolate structures of isoforms I, II and III. In this way 64 crystalline structures of the RbcL subunit of RuBisCO were downloaded, but redundant structures with missing amino acid residues were removed. These criteria allowed us to select 46 unique from wild-type RbcL and mutants in proteobacteria and archaea (Table S1 and Figure S1). The alignment of these amino acid sequences was performed with the MUSCLE algorithm [27]. All conformations were structurally superimposed on each other by least-squares fitting of the Cartesian coordinates of C-α atoms equivalent to the C-terminal domain, since this region was found to be the most structurally invariant. Principal component analysis (PCA) was used to evaluate the relationships between conformer sets of overlapping structures, as it is very useful for evaluating the distributions of experimental structures and comparing them with the conformations obtained through molecular dynamics (MD) simulations (Figure S1) [21,26].

### 2.2. RuBisCO Structure Selection and Phylogenetic Analysis

Based on the component analysis, RbcL^Wt^ structures and mutants from model organisms were selected which had a resolution ≤ 2.7 Å [28,29], which did not have missing amino acid residues, and for which the crystallized structure was ≥ 95% of the total protein (RbcL) in its three isoforms (I, II, III). Thus, 137 RuBisCO protein sequences, including the sequences provided by Kacar et al. [7] and the selected RuBisCO sequences, were used to build a phylogenetic tree according to Kacar et al. [7] with the PhyloBot web service [30]. RuBisCO orthologs were identified by the NCBI BLAST tool [25]. Then, multiple sequence alignments were inferred by the MSAProbs [31] and MUSCLE [27] algorithms with their default settings. Maximum likelihood (ML) phylogenetic inference was estimated using the PROTCATWAG model [32,33] in the RAxML web service [34]. Subsequently, ML phylogeny files were exported to the PhyML website [35] in order to calculate statistical support for branches as approximate likelihood ratios and the sequence from the group IV family as the outgroup to root the tree [7]. Finally, the phylogeny plot was developed with Mega6 software [36].

### 2.3. Normal Mode Analysis

Normal mode analysis (NMA) is a simple method to predict and characterize the internal dynamics of proteins, where slow low-frequency movements are often of functional importance [21,22]. NMA analyses were developed in the Bio3D package, where simultaneous analysis of a large set of structures is easily performed through the implementation of ensemble normal mode analysis (eNMA) [21,22], allowing the rapid characterization and comparison of flexibility across homologous structures. eNMA allows the prediction and identification of different flexibility patterns between different protein isoforms that are available at PDB [21,22]. In this way, high resolution crystallographic structures of the RbcL subunits of RuBisCO were selected: 6 structures of isoform I (PDB code: 1WDD, 4RUB, 5IU0, 1IWA, 1GK8 and 6FTL), 3 structures of isoform II (PDB code: 4LF1^WT^, 5HAN^S59F^ and 5HJX^A47V^) and 3 structures of isoform III (PDB code: 3A12^WT^, 3KDO^SP6^ and 3WQP^T289D^). As input, the set of pdbs structure aligned with MUSCLE software was provided [27]. Then, an efficient model based on C-alpha was used to enable the modes to be calculated quickly. Aligned eigenvectors and mode fluctuations were obtained as results for all RbcL structures.

### 2.4. Molecular Dynamics

The simulation models were built based on the high-resolution crystallographic structures of the RbcL subunits of RuBisCO; 6 structures of the isoform I were selected (PDB code: 1WDD, 4RUB, 5IU0, 1IWA, 1GK8 and 6FTL), as well as 3 structures of isoform II (PDB code: 4LF1^WT^, 5HAN^S59F^ and 5HJX^A47V^) and 3 structures of isoform III (PDB code: 3A12^WT^, 3KDO^SP6^ and 3WQP^T289D^). Before running MD simulations, proteins were treated. First, water molecules and monomers were eliminated. Moreover, missing amino acid residues from all structures were completed with MODELLER software version 10.0 (Accelerys, San Diego, CA, USA) [37]. The selected models satisfied spatial constraints such as bond lengths, bond angles, dihedral angles, and interactions between unbound residues. Models’ stereochemical quality was assessed with Ramachandran graphs generated on the MolProbity server [38], and fold quality was determined by Verify3D [39,40].

Molecular dynamics simulation was performed using the Groningen Machine for Chemical Simulations GROMACS version 2020 [41]. The PDB2GMX module was used to generate the topology that had information about the unbound parameters (types of atoms and charges) and bound parameters (bonds, angles and dihedrals) within the simulation. The CHARMM36 force field [42] was used for the simulations of all RuBisCO systems following similar studies [14,43,44]. Periodic boundary conditions (PBC) were applied in all directions of a cube box with a 10 Å lateral size. The systems were solvated with the TIP3P water model [36]. Na^+^ ions were added to neutralize the system, as in previous studies [45,46]. To minimize energy in all systems, the algorithm of descending steps was used with 50,000 steps and with a search for energy less than 1000 kcal/mol. We used the isothermal-isobaric set with two equilibrium phases to simulate a system at cellular physiological conditions. The first equilibrium phase was done in the NVT ensemble at a constant temperature of 300 K with a Berendsen thermostat. The second equilibrium phase was done in the NPT ensemble at a pressure of 1 bar for 2 ns with the Parrinello–Rahman barostat. The simulation was carried out for 50 ns with integration steps of 2 fs under constant pressure and temperature conditions with the leapfrog integration algorithm. The LINCS algorithm was used to constrain all bonds during equilibrium [47], and the Ewald particle mesh algorithm was used for long-range ionic interactions.

In the 50-ns MD simulation, 5000 trajectories were obtained. The analysis of the output structures was performed by the following GROMACS commands: gmx_mpi rmsd to calculate root mean square deviation (RMSD) values; gmx_mpi rmsf to calculate root mean square fluctuation (RMSF) values; and gmx_mpi gyrate to calculate the radius of gyration. Finally, PCA and DCCM analyses were carried out with the Bio3D package, following Yu and Dalby’s [48] recommendations. Conversion of the trajectory from XTC to DCD format was done with the CatDCD plugins of VMD software [49], and Pymol was used for image editing [50].

### 2.5. Stability and Flexibility Analysis

RMSD was used to measure the deviations of the protein backbone from its original structural conformation to its final structural conformation. When the stationary phase of the RMSD curve is reached, the protein is in equilibrium [51]. On the other hand, RMSF was used to measure the average individual residue flexibility during MD simulation. RMSF can indicate structurally which amino acids in a protein are more important in molecular motion [51]. RMSD and RMSF were performed using built-in protocols from GROMACS [41] and Bio3D [21,22].

### 2.6. Principal Component Analysis

Principal component analysis (PCA) was performed using the Bio3D package [21] implemented in the R-Project and ProDy software [23,24]. The PCA was carried out on Cα atoms during the last 40 ns of the trajectories [52]. The correlated movements of the whole protein can be represented by the eigenvectors and eigenvalues. The eigenvectors, also called principal components (PC), gave the direction of the coordinated movement of the atoms, and the eigenvalues represented the magnitude of the movement along the corresponding eigenvectors [53]. Thus, PC1 and PC2 were computed, because they contributed more significantly to the PCA analysis [54].

Briefly, the PCA was based on the diagonalization of the covariance matrix, *C*, with elements *Cij* calculated from the aligned and overlapping Cartesian coordinates, *r*, of equivalent Cα atoms [21]:

$$C_{ij}=\langle r_{i}-\langle r_{i}\rangle \;.\;\left(r_{j}-\langle r_{j}\rangle \right)\rangle$$

where *r_i_* and *r_j_* are cartesian coordinates of the *i*th and *j*th Cα atoms, and ⟨*r_i_*⟩ and ⟨*r_j_*⟩ represent the average time over all configurations derived from the molecular dynamics simulation. The analysis was limited to Cα atoms because they were less disturbed by statistical noise and offers a meaningful characterization of essential spatial movements [55].

### 2.7. Dynamic Cross-Correlation Matrices (DCCM)

To have a better understanding of the dynamics of the three RuBisCO isoforms, cross-correlation analysis (DCCM) was used to evaluate the motions (shifts) of alpha (Cα) carbon atoms in the *MD* simulations of all systems [56]. Additionally, it provides useful information regarding the mutation effect on protein dynamics by analyzing how atomic shifts were correlated [57,58], and it was constructed using the Bio3D package from R-Project [21].

The DCCM map is a 3D matrix annotation that displays time-related information for protein residues. Time-dependent data based on residuals can be analyzed using visual pattern recognition. The DCCM map shows the correlations of amino acid movements, and was calculated according to Ichiye and Karplus’ [59] equation:

$$C_{ij}=\frac{\left(\Delta r_{i}\;x\;\Delta r_{j}\right)}{{\left(\left\langle \Delta r_{i}{}^{2}\rangle \langle \Delta r_{j}{}^{2}\right\rangle \right)}^{1/2}}$$

where *Δr_i_* and *Δr_j_* are the displacements from the mean position of the *i*-th and *j*-th atoms with respect to time. The angle brackets “⟨⟩” represent the average time over the entire trajectory. *C_ij_* values ranged from −1 to +1; a positive value represented a positively correlated movement between residues *i* and *j*, while a negative value implied a negatively correlated movement between residues *i* and *j* [60,61].

## 3. Results

### 3.1. RuBisCO Forms Classification

**Figure 1:**
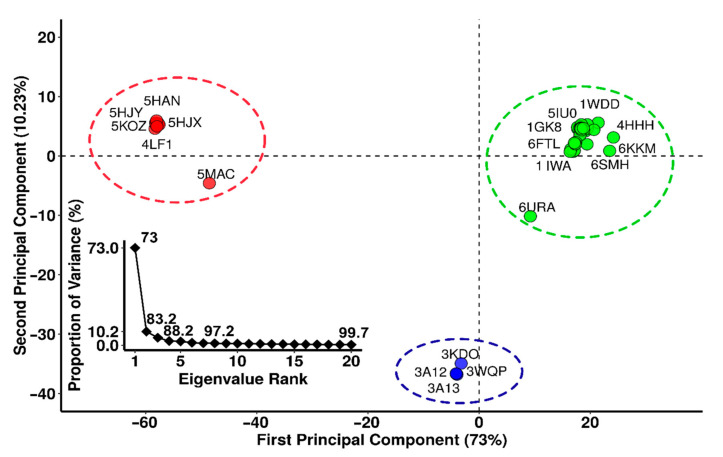
Principal component analysis (PCA) of RuBisCO RbcL isoforms. Green represents isoform I, red represents isoform II and isoform III is in blue.

From the RCSB protein database, 64 crystal structures of the RbcL subunit of RuBisCO were downloaded. A total of 18 structures were not considered due to a lack of coordinates, leaving 46 RuBisCO complexes between wild-types and mutants (Figure 1). Next, principal component analysis (PCA) was carried out. 73% of the total variance of the atomic fluctuations was captured along the first principal component (PC), while the second and third dimensions were necessary to capture 83.2 and 88.2, respectively (Figure 1).

The PCA structure shows three conformational clusters of RuBisCO (Figure 1). The largest cluster (in green) corresponds to 32 proteins (1BXN, 5OYA, 6FTL, 5MZ2, 5NV3, 1IWA, 1BWV, 5WSK, 2V6A, 1UW9, 2V68, 2VDH, 2VDI, 1GK8, 2V69, 4HHIH, 4RUB0, 4MKV, 1IR1, 1WDD, 3ZXW, 1RSC, 1RBL, 7JFO, 6URA, 1SVD, 1RLC, 1RLD, 1EJ7, 6SMH and 6KKM) and involves most RuBisCO structures from higher plants, green algae, blue-green algae, cyanobacteria, diatoms, and proteobacteria, including the 6URA structure of the bacteria *Promineofilum breve*, which is a benchmark to understand the evolution of RuBisCO Form I (Table S1). The second cluster (in red) includes 10 proteins (5MAC, 4LF1, 5HQM, 5KOZ, 5HQL, 5HJY, 5HAT, 5HAN, 5HAO and 5HJX), and they are mainly proteobacteria that present RuBisCO Form II, with the exception of 5MAC, which is found in *Methanococcoides burtonii* (archaea) and represents form II/III (Figure 1). Finally, in the third cluster (in blue), there are 4 proteins (3KDO, 3A13, 3WQP and 3A12), which corresponded to archaea (Figure 1).

Based on PCA analysis, RbcL wild-type structures and mutants from model organisms (Figure 1) were selected with a resolution ≤2.7 Å, with crystallized structure ≥95% and without any lost amino acid residues. This criterium allowed 6 wild-type structures to be selected from the largest cluster (in green: 1WDD, 5IU0, 4RUB, 1GK8, 6FTL and 1IWA). From the second cluster (in red) 1 wild-type structure was selected (4LF1^WT^) as well as 2 mutants (5HJX^A47V^ and 5HAN^S59F^), and from the third cluster (in blue) 1 wild-type (3A12^WT^) and 2 mutants (3KDO^SP6^ and 3WQP^T289D^) were selected.

**Figure 2:**
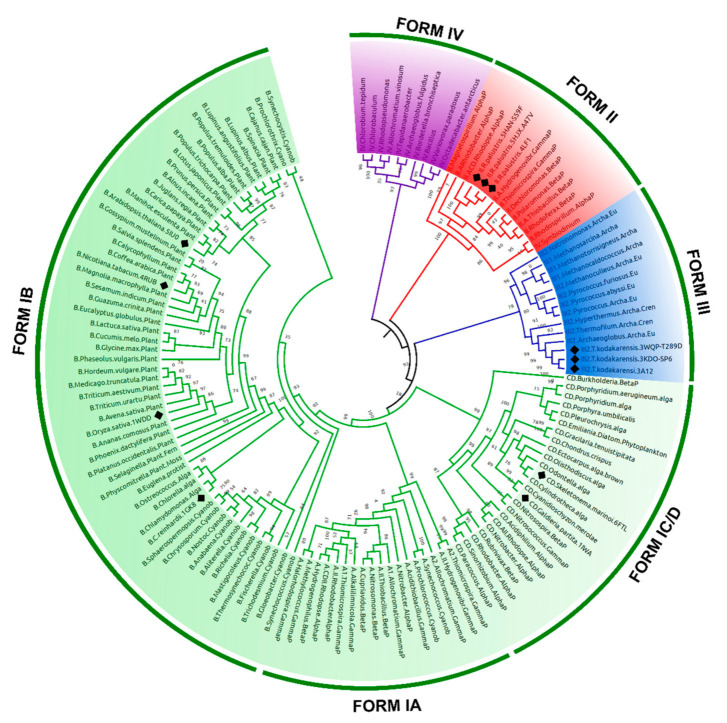
Maximum likelihood phylogenetic analysis of the RuBisCO RbcL protein family. Four forms of RuBisCO were classified along an evolutionary trajectory from the most recent common ancestor. Ancestral sequences were tagged according to their position in the RuBisCO subfamilies. Phylogenetic positions of the selected Wt and mutant proteins were marked with a black rhombus.

To classify the 12 selected structures according to their evolutionary groups, a maximum likelihood (ML) phylogenetic tree was made with 137 amino acid sequences of RuBisCO RbcL. The phylogeny shows that the different RuBisCO isoforms (I, II, III and IV) share a common evolutionary ancestor. The RbcL sequences of isoform I have subgroups IA, IB, and IC/D, which includes higher plants, cyanobacteria, green algae, red algae, and some bacteria. Consequently, the RuBisCO structures of the *Arabidopsis thaliana* (5IU0), *Nicotiana tabacum* (4RUB), *Oriza sativa* (1WDD) and *Chlamydomonas reinhardtii* (1GK8) species corresponded to IB subgroup (Figure 2), while the *Galdieria partita* (1IWA) and *Skeletonema marinoi* (6FTL) are related to the IC/D subgroup (Figure 2). The RbcL sequences of isoform II include proteobacteria and some eukaryotic alveolates; also within this clade are *Rhodopseudomonas palustris*, including members of this species which are wild-types (4LF1^WT^) and mutants (5HJX^A47V^ and 5HAN^S59F^) (Figure 2). Other wild-type (3A12^WT^) and mutant (3KDO^SP6^ and 3WQP^T289D^) structures belong to *Thermococcus kodakarensis*; its lineage arches and its RuBisCO structure are form III (Figure 2).

**Figure 3:**
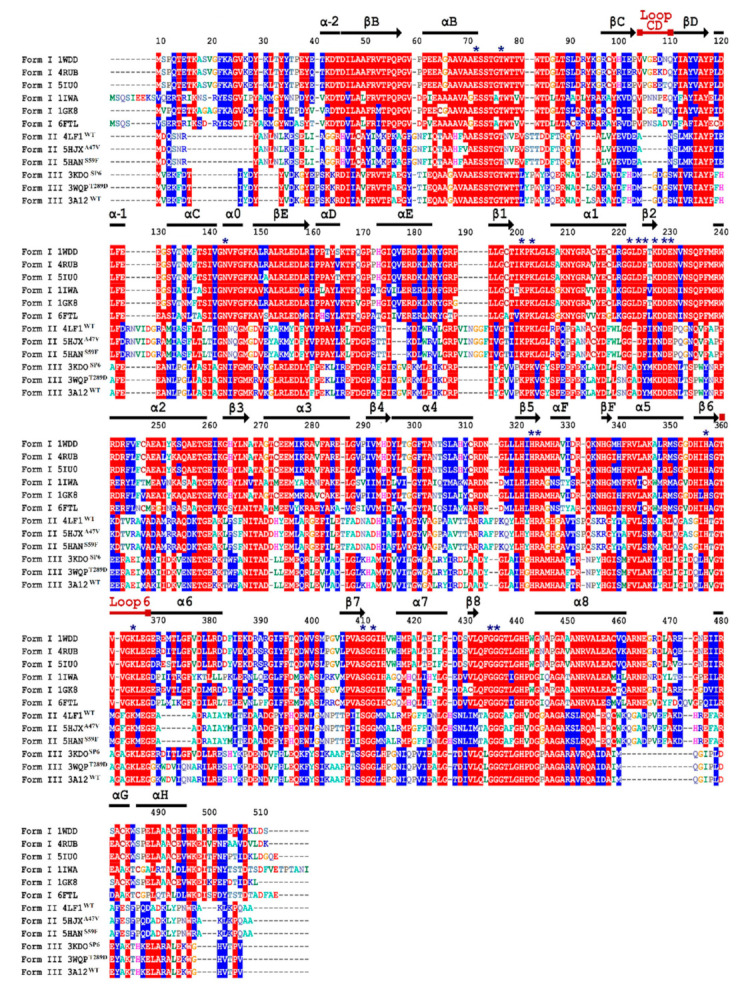
Multiple sequence alignment based on the structure of three isoforms of RuBisCO. Isoform I: *O. sativa* (1WDD), *N. tabacum* (4RUB), *A. thaliana* (5IU0), *C. reinhardtii* (1GK8), *G. partita* (1IWA) and *S. marinoi* (6FTL). Isoform II: 1 wild-type (4LF1^WT^) and 2 mutants (5HJX^A47V^ and 5HAN^S59F^) of *R. palustris*. Isoform III: 1 wild-type (3A12^WT^) and 2 mutants (3KDO^SP6^ and 3WQP^T289D^) of *T. kodakarensis*. Amino acid residue numbers are shown at the top of the sequence. Secondary structural elements, such as α-helices (bars) and β-strands (arrows), are shown in the figure. With an asterisk is depicted, it indicates mechanically important active site residues. Red background shading represents identical amino acids, blue shading designates similar amino acids while white shading indicates no similarity.

According to multiple sequence alignment analysis, the ends of the N-terminal and C-terminal regions showed greater variation in amino acid sequences. In Figure 3, the conserved active site residues are shown with an asterisk in the alignment. The secondary structures that maintain the amino acid residues involved in the catalysis were: αB (E72), α0 (N144), a loop that connects β1 and α1 (K202; K204), a catalytic motif located between α1 and α2 (G223; D225; F226; K228; D230; E231), β5 (H324) and loop 6 (K366) (Figure 3). Likewise, the secondary structures that are conserved and involved in the union of the phosphate groups in C1 and C5 of RuBP were: a loop between αB and βC (T77), β5 (R325), β6 (H358), β6, a loop that connects β7 and α7 (S411; G413) and a loop connecting β8 and α8 (G436; G437) (Figure 3).

The catalytic loop 6 in Figure 3 is characterized as a conserved and flexible sequence because it interacts with the tail in the C-terminal region to close on the catalytic pocket when it binds to RuBP. This tail then opens to allow product release. On the other hand, the CD loop is located in the N-terminal domain and approaches the opening of the active site from the opposite direction to loop 6; furthermore, it is packed against loop 6. The observed differences among RbcL sequences and between species is reflected in the molecular complexity of RbcL isoforms (Figure 3).

### 3.2. Stability and Flexibility Evaluation of RuBisCO Forms

**Figure 4:**
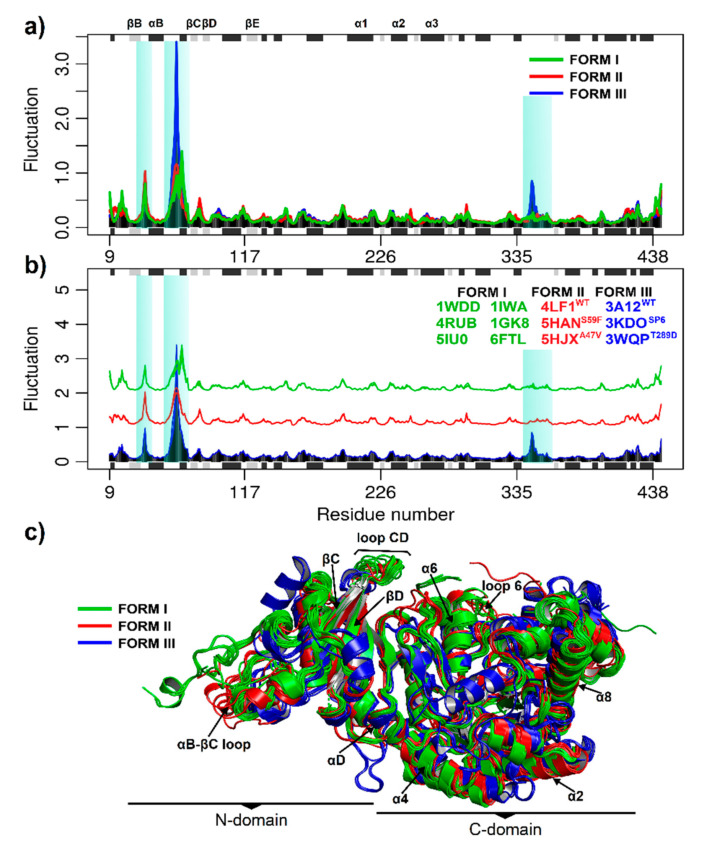
Normal mode analysis. (**a**) Consensus fluctuations of RuBisCO forms I, II and III. α-helices are in black and β-strands in gray; (**b**) Consensus fluctuations among species. In Form I are *O. sativa* (1WDD), *N. tabacum* (4RUB), *A. thaliana* (5IU0), *C. reinhardtii* (1GK8), *G. partita* (1IWA) and *S. marinoi* (6FTL). In form II are *R. palustris* with 1 wild-type (4LF1^WT^) and 2 mutants (5HJX^A47V^ and 5HAN^S59F^), and in form III are *T. kodakarensis* with 1 wild- type (3A12 ^WT^) and 2 mutants (3KDO^SP6^ and 3WQP^T289D^); (**c**) Monomeric structure of RbcL. The monomer is divided into two domains: the N-terminal domain and the C-terminal domain. Loop αB-βC, loop CD, loop 6 and α6 are indicated. The different colors indicate Form I (green), II (red), and III (blue).

Protein function depends on its structure and dynamics and can be altered by mutations. Consequently, it is necessary to understand the intrinsic structural flexibility of the observed differences in multiple sequence alignment. Thus, the flexibility of the RuBisCO forms was evaluated by normal mode analysis (NMA) and molecular dynamics (MD). In Figure 4, the eNMA shows the consensus fluctuations are highlighted and reveal a conserved pattern among species and RuBisCO forms (Figure 4a,b). The three isoforms show a greater fluctuation in the N-terminal domain spanning the amino acid residues (51–68) between the secondary elements αB and βC (Figure 4a), which are functionally relevant for RbcL. Likewise, RuBisCO form III presents greater fluctuation (≥3 Å) with respect to form I and II (Figure 4a,b). Conversely, the catalytic domain of the α/β barrel subunit (150–444) was more stable in all structures evaluated.

**Figure 5:**
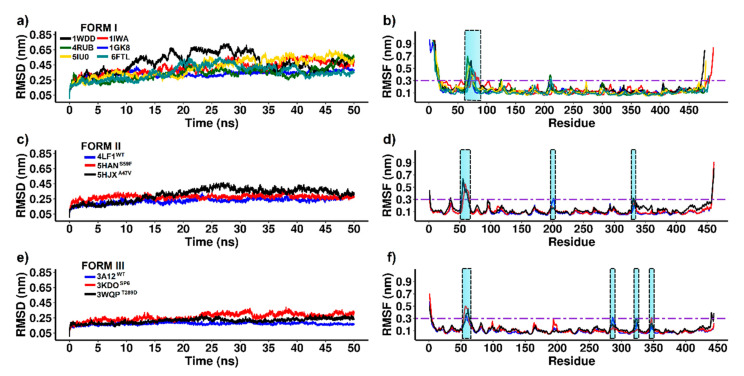
RMSD and RMSF profiles of RuBisCO forms I, II and III. (**a**) 50 ns RMSD of form I; (**b**) RuBisCO form I RMSF; (**c**) 50 ns RMSD of form II; (**d**) RuBisCO form II RMSF; (**e**) 50 ns RMSD of form III; (**f**) RuBisCO form III RMSF. RMSD was used to measure the deviations of the protein backbone from its original structural conformation to its final structural conformation. *C-alpha atoms* were used to calculate RMSF.

To evaluate the conformational changes of RuBisCO isoforms, we performed MD analysis for 50 ns over time. The mean square deviation (RMSD) was used to evaluate the conformational stability of the protein during the simulations. The mean square fluctuation (RMSF) was useful to identify rigidity and flexibility among RuBisCO forms. An RMSF value greater than 0.3 nm was considered as high fluctuation [62]. In Figure 5a, RSMD showed great variability in form I, where 4RUB (~0.36 nm ± 0.00268), 1GK8 (~0.34 nm ± 0.0025), 6FTL (~0.37 nm ± 0.0027) and 1IWA (~0.36 nm ± 0.0032) presented the lowest values (Table 1). Contrary to this, 1WDD (~0.52 nm ± 0.004) and 5IU0 (~0.46 nm ± 0.0033) showed very high RMSD values.

The RMSF of form I showed greater fluctuations in the N-terminal and C-terminal tails because the first and last amino acid residues form a loop-shaped structure. Likewise, the six systems showed greater flexibility in the loop located between αB and βC (≥0.3 nm) of the N-terminal domain spanning the following amino acid residues: 1WDD (Thr68−Ser76), 4RUB (Gly64−Thr75), 5IU0 (Trp66−Thr75), 1IWA (Ala73−Ala86), 1GK8 (Val69−Thr75), and 6FTL (Ser71−Thr80); see Table 1. On the other hand, the active site of RuBisCO (α/β barrel) was stable (RMSF ≤ 0.3 nm) because it is located in the TIM barrel domain that allows a protein to be slightly rigid (Figure 5b).

On the other hand, the analysis of form II included the 4LF1^WT^ system and two mutants, 5HAN^S59F^ and 5HJX^A47V^. The results showed that 4LF1^WT^ (~0.24 nm ± 0.001) and the mutant 5HAN^S59F^ (~0.28 nm ± 0.002) had lower RMSD values and greater stability during the trajectories. Moreover, there was an increase in the RMSD value of the 5HJX^A47V^ mutant (~0.34 nm ± 0.002), and this can be attributed to the presence of valine 47 (A47V) in the αB region at the N-terminal end (Table 1). On the other hand, the 4LF1^WT^ structure and mutants (5HAN^S59F^ and 5HJX^A47V^) had similar flexibility in most amino acid residues. Among the systems analyzed in form II, the greatest fluctuation was found in the loop between αB and βC (4LF1^WT^ (Gly53−Asp63 residues), 5HAN^S59F^ (Val56−Thr65 residues) and 5HJX^A47V^ (Thr54−Asp63 residues)), and other relevant fluctuations (≥0.3 nm) were: 4LF1^WT^ (Val201−Phe202, Pro458−Ala461 residues), 5HAN^S59F^ (Pro458−Ala461 residues) and 5HJX^A47V^ (Lys330−Met331, Pro458−Ala461 residues). In conclusion, the 5HAN^S59F^ mutant analysis allowed key residues that reduced (Gly53−Glu57 residues, Val201−Phe202 residues) and increased (Phe59, Asp63−Phe64 residues) the flexibility by ≥0.1 nm to be identified (Table 1). Changes were found at loop 6 and in the loop between αB and βC, which is a critical region for gaseous substrate binding after RuBP enolization has been completed [18,63]. Moreover, the comparison between 4LF1^WT^ and 5HJX^A47V^ allowed the identification of key residues that increased (Gly35, Lys330−Met331) and reduced (Val201−Phe202) RuBisCO form II fluctuation by more than 0.1 nm (Table 1), which can be attributed to A47V mutation and which has an effect on RuBisCO catalytic activity [63].

The RMSD analysis of form III showed that the 3A12^WT^ protein (~0.18 nm ± 0.001) was more stable than 3KDO^SP6^ (~0.28 nm ± 0.001) and 3WQP^T289D^ (~0.22 nm ± 0.001); see Table 1. The 3KDO^SP6^ mutant showed regions where RMSF differed markedly from WT (Figure 5e). The residues Trp55-Tyr62 (loop that connects αB and βC) and Asn347 (loop that connects α6 and β7) showed, on average, a higher RMSF than WT (Table 1). However, the region that exhibited a lower RMSF in the 3KDO^SP6^ mutant with respect to WT was the αF region (Ala286); see Table 1. Moreover, the analysis of the 3WQP^T289D^ mutant showed a fluctuation greater than 0.1 nm in residues 60–61 (loop connecting αB and βC) and 322 (loop 6); see Table 1. Likewise, amino acid 322 is involved in direct interaction with the ligand CAP 2-carboxyarabinitol-1,5-diphosphate (C_6_H_14_O_13_P_2_). Thus, loop 6 is a region that plays a critical role in improving the enzymatic activity in the 3WQP^T289D^ mutant. Finally, our results of NMA and RMSF are in agreement because they were able to identify similar regions of greater flexibility in RuBisCO isoforms, where the loop between αB and βC presented greater flexibility.

### 3.3. Principal Component Analysis (PCA)

**Figure 6:**
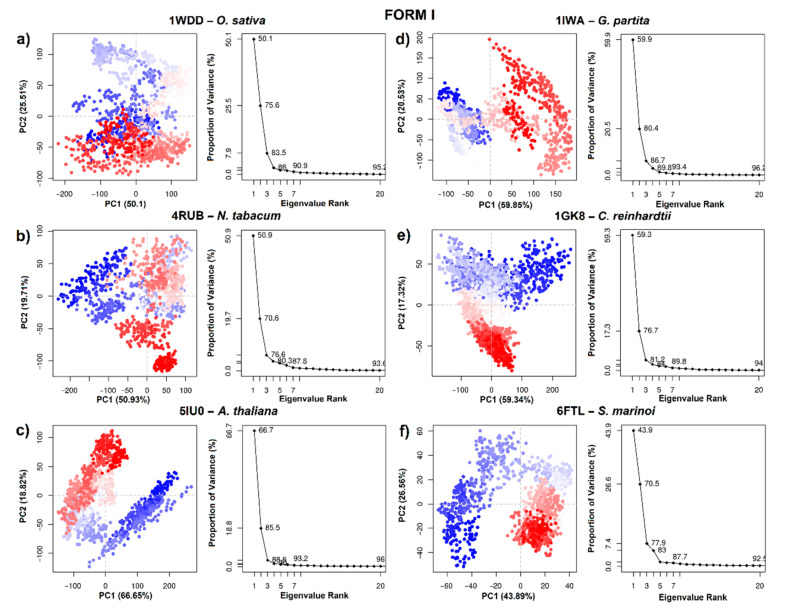
Principal component analysis of RuBisCO isoform I. (**a**) *O. sativa* (1WDD); (**b**) *N. tabacum* (4RUB); (**c**) *A. thaliana* (5IU0); (**d***) G. partita* (1IWA); (**e**) *C. reinhardtii* (1GK8) and (**f**) *S. marinoi* (6FTL).

To obtain information on the conformational states of RbcL form I (5IU0, 1IW1, 1GK8, 1WDD, 4RUB and 6FTL), the PCA of the Cα atoms was carried out. The first two PCs (PC 1/2) were taken in account. Figure 6 indicates the variance in the conformational distributions of proteins, where the display of continuous color points (from blue to white and to red) highlights periodic jumps between structural conformations. Moreover, the PC 1/2 of the MD trajectories were quite varied for the six systems, showing differences in the movement and stability of RuBisCO form I (Figure 6). In 5IU0, 1IWA, 1GK8 and 1WDD systems, there was greater correlated movement along the first two components, with a percentage of 85.5%, 80.4%, 76.7% and 75.6%, respectively, while in the 4RUB and 6FTL systems, the PC values were 70.6% and 70.5%, respectively (Figure 6). On the other hand, the PC 1/2 for 5IU0, 1GK8, 1IWA and 6FTL systems clearly shows the thermodynamically distinct periodic jumps (Figure 6), where most of the blue and red dots were assembled and distributed in opposite regions; therefore, proteins were in a relatively stable state in the system (Figure 6). Thus, the RbcL structures of *Oryza sativa* (1WDD) and *Nicotiana tabacum* (4RUB) showed a uniform distribution, overlapping PC subspace where there were not energy barriers, because most dots were in a scattered state. PCA analysis could suggest that the IB substructure of RbcL may undergo a periodic change in its conformation to reorient its domains (N-terminal and α/β barrel).

**Figure 7:**
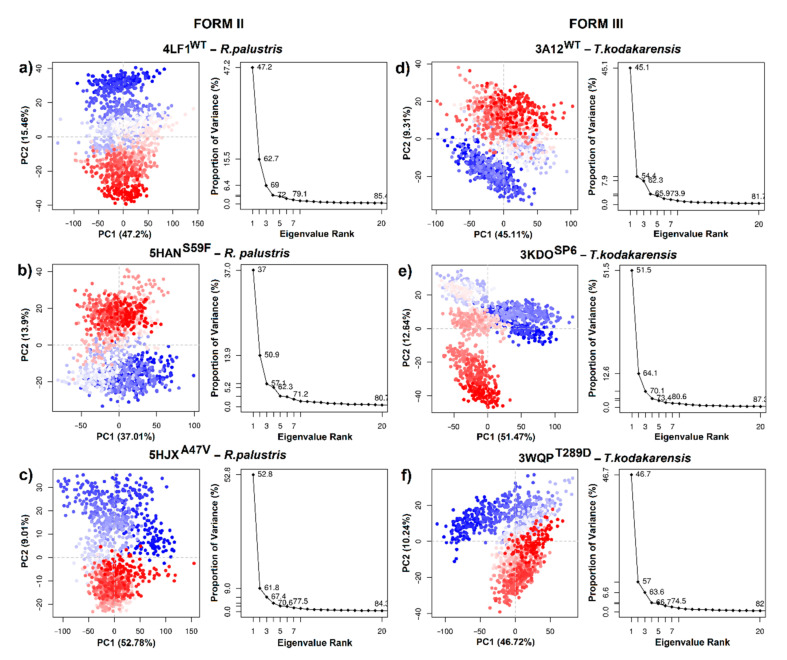
Principal component analysis of RuBisCO isoforms II (*R. palustris*) and III (*T. kodakarensis*). (**a**) 4LF1^WT^ (*R. palustris*); (**b**) 5HAN^S59F^ (*R. palustris* mutant); (**c**) 5HJX^A47V^ (*R. palustris* mutant); (**d**) 3A12^WT^ (*T. kodakarensis*); (**e**) 3KDO^SP6^ (*T. kodakarensis* mutant); and (**f**) 3WQP^T289D^ (*T. kodakarensis* mutant).

Regarding RuBisCO form II, PCA analysis allowed information on the conformational states of 4LF1^WT^ and two mutants (5HAN^S59F^ and 5HJX^A47V^) to be obtained. The PC 1/2 of 4LF1^WT^, 5HAN^S59F^ and 5HJX^A47V^ was 62.66%, 50.91% and 61.79%, respectively (Figure 7a–c). The scatter distribution of red and blue dots represents two different stable conformational states of the protein. The 4LF1^WT^ system was revealed to be more stable than the mutants (5HAN^S59F^ and 5HJX^A47V^). Finally, the scatter plot of the 5HJX^A47V^ mutant (Figure 7c) showed the most unstable state of *R. palustris*. This is in agreement with RMSD results (Figure 5c), where 5HJX^A47V^ demonstrated more flexibility (~0.1 nm) than WT. Thus, the more dispersed conformational state was produced by repressor mutations (S59F and A47V).

Figure 7 shows PCA analysis of *T. kodakarensis* with 1 wild-type (3A12^WT^) and 2 mutants (3KDO^SP6^ and 3WQP^T289D^). The first two eigenvectors captured most of the variance. The PCs (PC1/2) of the three systems contributed 54.42%, 64.11% and 56.96%, respectively (Figure 7d–f). Likewise, the analysis of the variance in the conformational distribution of 3KDO^SP6^ and 3WQP^T289D^ shows that mutants were energetically more stable than the WT system (Figure 7d–f). This analysis suggests that the WT may undergo a periodic change in its conformation to reorient its N-terminal or C-terminal domain. Differentiated grouping can be energy expensive; however, it can provide a control mechanism in the photosynthetic activity of RuBisCO.

### 3.4. Dynamic Cross-Correlation Matrix (DCCM)

**Figure 8:**
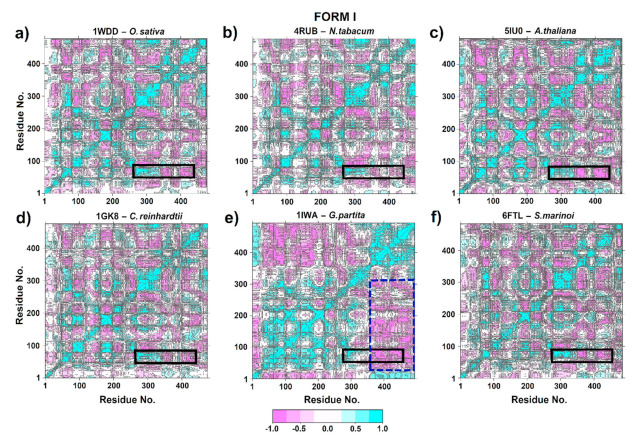
Cross-correlation analysis (DCCM) of RuBisCO form I. (**a**) *O. sativa* (1WDD); (**b**) *N. tabacum* (4RUB); (**c**) *A. thaliana* (5IU0); (**d***) G. partita* (1IWA); (**e**) *C. reinhardtii* (1GK8) and (**f**) *S. marinoi* (6FTL). The color scale ranges from pink (for values ranging from −1 to −0.5) to white (−0.5 to 0.5) and to cyan (0.5 to 1).

One structural transition that is essential for the carboxylation of the 2,3-ene-diol(ate) intermediate is the closure of the active site of loop 6 in the large subunit and the concomitant movement of loop connecting αB and βC at the N-terminal end to stabilize the catalytic of loop 6 conformation [18,63,64]. Therefore, DCCM was performed to probe the conformational ensemble of these zones. Thus, similar correlation patterns were observed in 1WDD, 4RUB, 5IU0, 1GK8 and 6FTL (Figure 8a–d). This may provide insights into a conserved mechanism among *Chlamydomonas*, *Skeletonema*, *Arabidopsis*, tobacco and rice, since there was a correlated movement of residues 60–80 (part of αB region and the loop connecting αB and βC) at the N-terminal end with secondary structures as α4, β5, αF, βF, α5, β6 and loop 6, which are located between residues 270–345 in the C-terminal end (Figure 8a–d). This correlation is important in RuBisCO since it connects the region with the greatest flexibility (loop connecting αB and βC) with the key substrate-binding residues H294, R295, H327 and K334. Moreover, the βC, loop CD, βD and αC structures had a strong negative correlation with α4, β5, αF, βF, α5, β6 and loop 6 structures. This suggests highly synchronized movements of the RuBisCO structure.

On the other hand, *Galdieria partita* (1IWA) had a different correlation pattern than other isoform I proteins (Figure 8e). Thus, 1IWA presented a strong negative correlation (≥−0.5) at residues 25–220 (secondary structures α-2, βB, αB, βC, loop CD, βD, α-1, αC, α0, βE, αD and αE) with respect to amino acid residues located at position 350–493 (α6, β7, α7, β8, α8, αG and αH) in the blue dotted line rectangle. However, a positive correlation was observed between the loop that connects αB and βC with the structures β4, α4, β5, αF, βF, α5, β6 and loop 6 (black rectangle); see Figure 8e.

**Figure 9:**
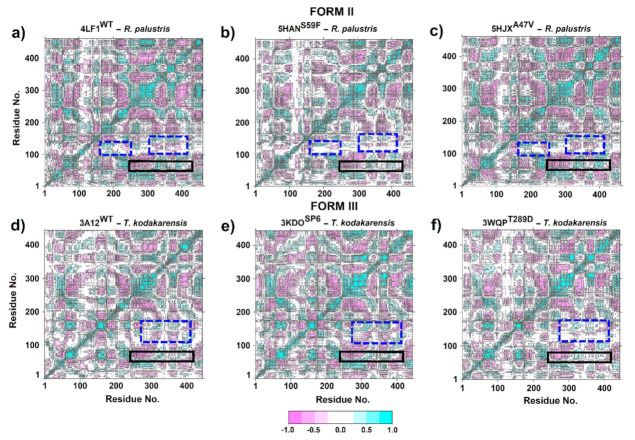
Cross-correlation analysis (DCCM) of RuBisCO form II (*R. palustris*) and III (*T. kodakarensis*). (**a**) 4LF1^WT^ (*R. palustris*); (**b**) 5HAN^S59F^ (*R. palustris* mutant); (**c**) 5HJX^A47V^ (*R. palustris* mutant); (**d**) 3A12^WT^ (*T. kodakarensis*); (**e**) 3KDO^SP6^ (*T. kodakarensis* mutant); and (**f**) 3WQP^T289D^ (*T. kodakarensis* mutant). The color scale ranges from pink (for values ranging from −1 to −0.5) to white (−0.5 to 0.5) and to cyan (0.5 to 1).

The DCCM plots for RuBisCO Form II showed that residues 53–63 exhibited anticorrelated movement with the structures β4, α4, β5, αF, βF and α5 (Ala255–Gly317). Moreover, the most flexible region (residues 53–63) located at the loop connecting αB and βC moves in the same direction (positive correlation > 0.5) as β6, loop 6 and α6 structures that are located between residues Gly326-Ala341 at the C-terminal domain (Figure 8a–c). This is because the movement of the N-terminal domain (the loop that connects αB and βC) towards the active site is important, since it is a key step in the catalytic mechanism of RuBisCO that involves CO_2_ addition. Furthermore, the comparison between 5HAN^S59F^ (Figure 9b) and LF1^WT^ (Figure 9a) showed minor anticorrelations (blue dashed line box). Therefore, S59F mutation is the most flexible region with a direct effect on loop 6 residues. Regarding the 5HJX^A47V^ mutant (Figure 9c), no significant changes were found in relation to 4LF1^WT^ (Figure 9a).

The DCCM analysis of the loop connecting αB and βC in RuBisCO form III demonstrated an anticorrelated movement (<−0.5) of Pro60-Ala72 residues with β6 and loop 6 structures. However, Ser50-Tyr59 residues presented a positive correlation (>0.5) with respect to β6 and loop 6 structures (Figure 9). On the other hand, correlated movements were significantly reduced in 3WQP^T289D^ with respect to 3A12^WT^ (blue dotted line rectangle). This region comprises a positive correlation between αE, β1 and loop structures that connect β1 and α1 with the β6 and loop 6 structures. However, the positive correlation movements of these regions decreased in the 3WQP^T289D^ mutant (Figure 9f), since loop 6 (Lys322) mutation affects the correlation between specific residues, making them more flexible. Finally, the 3KDO^SP6^ mutant (Figure 9e) did not show visible changes in the residue-residue correlation patterns when compared to 3A12^WT^ (Figure 9d).

## 4. Discussion

RuBisCO plays a key role in carbon fixation on Earth. This enzyme possibly evolved during the Archean Eon [65] from an ancestral non-carbon fixing enzyme long before the appearance of the Calvin–Benson–Bassham cycle [3,66]. Thus, our phylogenetic analysis (Figure 2) allows the different RuBisCO isoforms to be identified, supporting the idea that photosynthetic RuBisCO (form I, II and III) and RubisCO-like protein (RLP, Form IV) evolved from the same ancestral protein [67,68]. This would have allowed photosynthetic organisms to adopt different strategies to improve CO_2_ specificity (as in the *Galdieria partita* case) [69], increase intracellular CO_2_ concentration through a mechanism of carbon concentration [70], or inhabit ecological niches that have low levels of O_2_/CO_2_, such as in methanogenic organisms (as in the *Methanococcoides burtonii* case) [66].

The RbcL subunit is common among all isoforms (Figure 1). Currently, there are more than 64 RbcL structures in the PDB database (Figure 1, Table S1). Our PCA results using the Bio3D package [21] to identify three clusters with different structural flexibilities (Figure 1). This also allowed the identification of the key amino acid residues, several structural characteristics, and conformational changes that are critical for folding and catalytic activity (Figure 3). The largest cluster corresponded to Isoform I, which included higher plants, green algae, blue-green algae, cyanobacteria, diatoms, and proteobacteria (Figure 1). The emergence of form I complexes through the incorporation of small subunits represents a transitional key that is little understood in RuBisCO evolution [19]. However, the 6URA structure of *Promineofilum breve* (a bacteria) has a structural flexibility that allowed grouping of isoform I (Figure 1), taking into account that 6URA does not present small RbcS subunits [71] and also presents deletions in secondary structural elements such as loop-CD and the loop that connects α8-αG, making it a reference point to advance our understanding of isoform I evolution. In the second cluster (Figure 1), proteobacteria of RuBisCO Form II were mostly reported, except for the methanogenic archaea *Methanococcoides burtonii* (5MAC), which presents an isoform II/III [5,65]. It also shows a unique insert of 26–30 amino acids between the α6 and β7 secondary structures at the bottom of the βα-barrel [72]. Methanogens like *Methanococcoides burtonii* (5MAC) are strictly anaerobic and cannot survive in the presence of oxygen [73]. Thus, their RuBisCO are not under selection pressure to mitigate the competitive binding of O_2_ over CO_2_.

Despite having only 30% amino acid identity, the multiple sequence alignment analysis among RuBisCO isoforms showed large changes in the N-terminal and C-terminal regions (Figure 3). Moreover, the structures retain the residues involved in substrate binding and catalytic activity (Figure 3), supporting the idea that they are critical to folding and maintaining the overall structure and function of the photosynthetic RuBisCO [1,66]. Moreover, with the molecular dynamics analysis, it was possible to sample the transition of the RuBisCO conformation in 50 ns, and the RMSD results were consistent with previous studies [14,45,46]. Our results added more theoretical evidence on the structural movements of RuBisCO, helping to understand the structure flexibility and how this could affect the synchronization of the residues and the closing mechanism, which are still unknown [19]. Thus, the NMA and RMSF results revealed similar flexibility patterns in the RbcL structures (Figure 4 and Figure 5), showing the distribution of temperature factors (B-factors) and the fact that loops which are more flexible during catalysis are also more flexible in crystallized structures [74,75,76]. Consequently, our results indicate that secondary structures such as the loop connecting αB and βC (~64–85) and the tails in the N-terminal and C-terminal region show greater fluctuations in the three isoforms (Figure 5), and the structural movements are related to the structural changes and activities of RuBisCO during the transition from its open to closed state [74,77]. Thus, closed-state carboxylation of RuBisCO is more likely when the substrate is attached, whereas fluctuations in larger tails can also cause structural changes and deactivations of RuBisCO.

According to DCCM analysis of isoform I, the structure of 1WDD, 4RUB, 5IU0, 1GK8 and 6FTL preserved the direction of the movements in the time of residues ~64–85 (part of the αB region and the connecting loop between αB and βC) and ~270–345 residues (related to the secondary structures α4, β5, αF, βF, α5, β6 and loop 6); see Figure 8. This correlation is important for RuBisCO activity, since it connects the region of greatest flexibility (connecting loop between αB and βC) with the key residues (H294, R295, H327 and K334) that bind the substrate (Figure 4), where the two states (open and closed) of RuBisCO are distinguished by the degree of accessibility of the solvent to the active site [74]. The closed state is associated with substrates and inhibitors (CABP 2-carboxyarabinitol 1,5-bisphosphate) that are attached to the active site. This would be achieved through a movement of loop 6 (residues 331–338) and the connecting loop between αB and βC (residues ~64–85); see Table 1. In addition, the N-terminal and C-terminal loops function like latches that hold loop ~64–85 and loop 6 in their closed positions with an extremely slow release of CABP [74]. Moreover, RuBisCO studies carried out on spinach and wheat were able to show that residues ~8 to 20 at the N-terminal end are only ordered when the active site is closed [78]. In the closed conformation, the N-terminal end (Phe13, Lys14, Gly16 and Lys18) is placed directly on the connecting loop between αB and βC (~64–85 residues), which coordinates the P1 site of the substrate [78]. In contrast, the open state is associated with weak products being attached (metal ions that are catalytically inert). In the open state, loop 6 of the α/β barrel is away from the active site, and the C-terminal end of the large subunit is disordered, so the active site is open.

On the other hand, *Galdieria partita* is a thermophilic red alga with a high specificity, and this alga showed a marked difference with respect to the RuBisCO of higher plants [79]. Our results are in agreement with Watanabe et al. [79] because the movements of the RbcL structure of *G. partita* presented a different residue-residue correlation pattern than IB and IC/D structures (Figure 8e). Although *G. partita* has more residues in the N-terminal and C-terminal regions (Figure 3), this phenomenon could play an important role in the structural movement of the RuBisCO enzyme, as indicated by some studies [78]. Likewise, the RMSF analysis of 1IWA (*G. partita*) showed the greatest flexibility between residues ~Trp73–Ala86 (the connecting loop between αB and βC). Moreover, it is necessary to point that Thr74 in the RbcL sequence of *G. partita* is an evolutionarily conserved amino acid that binds the phosphate groups of RuBP. Thus, the Thr74 residue can alter the packing in the C-terminal end [79]. Likewise, when RuBisCO is in the open state, the hydrogen bond breaks between P1 and Thr74, as well as between Thr76 and Trp470 [79]. Thr74 is stabilized by the carbonyl of the Thr76 backbone, while the stabilization of the Thr76 and Trp470 side chains occurs in the closed state of loop 6 [79]. Moreover, the work of Satagopan et al. [63] on *R. rubrum* (RuBisCO isoform II) is in agreement with Watanabe et al. [79], because the link between the αB and βC loop with loop 6 was also identified; this link allows the conformation of the catalytic loop to be stabilized. Satagopan et al. [63] used mutants (5HAN^S59F^ and 5HJX^A47V^) in *R. rubrum* where CO_2_ was the only carbon source; their results showed a similar biological growth between mutants and a decrease with respect to WT [63,80]. Thus, our PCA results of DM indicated that mutants (5HAN^S59F^ and 5HJX^A47V^) would have undergone a change from an unstable intermediate conformation (4LF1^WT^) to an unstable one, showing a greater variation in the conformational distribution of the RuBisCO (Figure 7b,c), being consistent with the RMSD results where 5HJX^A47V^ was more flexible (~0.1 nm) than WT (Figure 5). Consequently, the fluctuation in the 5HJX^A47V^ mutant was increased by more than 0.1 nm in the key residues (Lys330–Met331) that are located in loop 6 (Table 1). Some experimental studies showed that the mutations (M331L and M331A) affected the growth of the purple photosynthetic bacterium *R. palustris*.

Thus, the residue Met331 and its interactions seem to be specific and critical for the addition of CO_2_ to the intermediate 2,3-enediol(ate) derived from RuBP [63,80]. On the contrary, strains with double mutations (A47V/M331A and S59F/M331A) in *R. palustris* did show growth. Thus, growth inhibition induced by M331A was suppressed by the substitution of A47V and S59F [63]. To explain this phenomenon, Satagopan et al. [80] evaluated the movements and crystallized structures of WT and mutants, where the α carbon of Ala47 (structure αB) was ∼15 Å away from Met331 (loop 6) [80]. Furthermore, our results indicate that Ala47 and Met331 move in the same direction (positive correlation > 0.5; Figure 9a–c). Likewise, the side chain is found in a hydrophobic environment within 4 Å of Ala70 and Val72 [80]. Substitution with a Val (A47V) would push the αB structure towards the active site, and thus Lys330 and Met331 showed greater flexibility (Table 1; Figure 5). Glu49 located in the αB structure works to stabilize Lys330 and helps to close loop 6 during catalysis, and Thr54 binds phosphate P1 of the substrate [78,81,82].

*Thermococcus kodakarensis* is a hyperthermophilic archaea. Its optimal growth is at 85 °C, and it can exceed the activity of RuBisCO spinach by 20 times [12,83], but at room temperature its activity is only one-eighth [12]. For this reason, it is necessary to develop new *T. kodakarensis* strains with good photosynthetic performance at room temperatures. In this sense, 3WQP^T289D^ [12] and 3KDO^SP6^ [10] mutants were developed. Their results show an increase in the carboxylase activity of 24% and 31%, respectively. In the 3KDO^SP6^ mutant, residues of the α6 region were replaced by 11 amino acid residues from spinach (E326-L336 “ERDITLGFVDL”). Consequently, Nishitani et al. [10] evaluated the flexibility between 3A12^WT^ and 3KDO^SP6^, showing that there is an increase in temperature factors (B-Factors Å2) in the secondary structure α6. Moreover, our ANM results showed a greater fluctuation in the loop between αB and βC and in the loop that connects α6 and β7 (His341-Ala361) with respect to isoforms I and II. Likewise, the mutants were more energetically stable than WT (Figure 7e,f), suggesting that WT may undergo a periodic change in its conformation to reorient its N-terminal or C-terminal domain, since the differentiated clustering of RuBisCO conformational distributions can be energetically expensive [84]. However, it can also provide a control mechanism in the photosynthetic activity of RuBisCO.

In addition, 3KDO^SP6^ showed greater flexibility of ~0.15 nm in the loop that connects αB and βC (Figure 5f) and the 3WQP^T289D^ mutant in the residue Lys322 (a residue of loop 6 which is catalytically critical) (Figure 5f, Table 1). From this analysis, the changes between αF and βF in 3WQP^T289D^ can influence loop 6′s movement (Table 1). Therefore, an increase in the loop flexibility between αB and βC, Lys322 or residues in the vicinity of the catalytic center is important to increase the catalytic activity of RuBisCO from *T. kodakarensis* at room temperature. Finally, our MD simulation results corroborate very well the work of Satagopan et al. [63] and Fujihashi et al. [12]. Likewise, the regions of greater flexibility and active sites exhibit highly correlated or anticorrelated movements between the different isoforms (Figure 8 and Figure 9), building a dynamic correlation network where information signal are transmitted [48]. Therefore, it is necessary to build next-generation computational tools, where a PAN-DM approach will allow us to integrate very complex information on molecular structure, dynamics and evolution.

## 5. Conclusions

Our PCA showed a wide range of the conformational space of the RuBisCO crystal structures, allowing the identification of different isoforms. Likewise, phylogenetic analysis supports the idea that RuBisCO evolved from the same ancestral enzyme, conserving the residues involved in substrate binding and catalytic activity. On the other hand, molecular dynamics analyses were able to sample the transition of RuBisCO conformation in 50 ns. Thus, the NMA and RMSF results revealed similar flexibility patterns. The places where the secondary structures loop between αB and βC as well as the tails in the N-terminal and C-terminal regions show greater fluctuations among the three isoforms, and their movements are possibly related to the structural changes and functional activities of the RuBisCO enzyme during the transition from its open to closed state. On the other hand, the DCCM results indicate that there are changes in the movement direction of the secondary structures of the three isoforms. However, movements in the same direction are preserved in loop 6 and the connecting loop between αB and βC. This correlation is important for enzymatic activity and to stabilize the conformation of the catalytic loop. In isoform I, the 1WDD, 4RUB, 5IU0, 1GK8 and 6FTL structures showed a positive correlation between the residue movement of ~64–85 (part of the αB region and the connecting loop between αB and βC) and residues ~270–345 (secondary structures α4, β5, αF, βF, α5, β6 and loop 6). On the other hand, 1IWA (*Galdieria partita*) had a marked difference in the direction of structural movements with respect to the others’ isoform I. This could have a key role, and it is required to deepen its study with mutants. On the other hand, the PCA results of *R. rubrum* (RuBisCO form II) indicated that mutants (5HAN^S59F^ and 5HJX^A47V^) would have undergone a change from an unstable intermediate conformation (4LF1^WT^) to an unstable one, showing a greater variation in the conformational distribution of RuBisCO. Consequently, the 5HJX^A47V^ mutant allowed the fluctuation of the key residues Lys330–Met331 in loop 6 to be increased by more than 0.1 nm. Regarding isoform III, the mutants (3KDO^SP6^, 3WQP^T289D^) of the hyperthermophilic archaea *Thermococcus kodakarensis* were more energetically stable than WT, suggesting that the WT may undergo a periodic change in its conformation to reorient its N-terminal or C-terminal domain, a control mechanism in the photosynthetic activity of RuBisCO. 3KDO^SP6^ showed a greater flexibility of ~0.15 nm in the loop between αB and βC and Lys322 in the 3WQP^T289D^ mutant (a catalytic residue critical in loop 6) with respect to 3A12^WT^. Thus, an increase in the loop flexibility between αB and βC, Lys322 or residues in the vicinity of the catalytic center is important to increase the photosynthetic efficiency of RuBisCO from *T. kodakarensis* at room temperature. Finally, our results added more evidence regarding the structural movements of RuBisCO, helping to understand the details of the synchronization and the closing mechanism that are still unknown.

## Figures and Tables

**Table 1:** Average and standard error of RMSD in 12 RbcL structures. Regions were chosen according to their residues with the highest mean RMSF (≥0.3 nm).

Form	Protein	RMSD	Region ≥0.3 of RMSF	Sequence	Structures
I	1WDD^WT^	0.52 ± 0.004	68–76	TVWTDGLTS	Loop connecting αB and βC
	4RUB^WT^	0.36 ± 0.002	64–75; 125; 209–211	GTWTTVWTDGLT; F; QPF	Loop connecting αB and βC; α0; Loop connecting β2 and α2
	5IU0^WT^	0.46 ± 0.003	22; 66–75	L; WTTVWTDGLT	N-terminal tail; Loop connecting αB and βC
	1IWA^WT^	0.44 ± 0.003	55–56; 73–86; 482	PG; WTVVWTDLLTAA; T	βB; Loop connecting αB and βC; C-terminal tail
	1GK8^WT^	0.34 ± 0.002	69–75	VWTDGLT	Loop connecting αB and βC
	6FTL^WT^	0.37 ± 0.002	71–80; 211–212	TVVWTDLLTA; NS	Loop connecting αB and βC; Loop connecting β2 and α2
II	4LF1^WT^	0.24 ± 0.001	53–63; 201–202	GTNVEVSTTDD; VF	Loop connecting αB and βC; Loop connecting β2 and α2
	5HAN^S59F^	0.28 ± 0.002	56–65	VEVFTTDDFT	Loop connecting αB and βC
	5HJX^A47V^	0.34 ± 0.002	54–63; 330–331	TNVEVSTTDD; KM	Loop connecting αB and βC; Loop 6
III	3A12^WT^	0.18 ± 0.001	58–59; 286	LY; A	Loop connecting αB and βC; αF
	3KDO^SP6^	0.28 ± 0.002	55–62; 347	WTTLYPWY; N	Loop connecting αB and βC; Loop connecting α6 and β7
	3WQP^T289D^	0.22 ± 0.001	57–63; 322	TLYPWYE; K	Loop connecting αB and βC; Loop 6

## Data Availability

The datasets generated and/or analyzed during the current study, are available on request from the corresponding author.

## Supplementary Materials

The following are available online at https://www.mdpi.com/article/10.3390/biom11121761/s1, Table S1: 46 structures of isoforms I, II and III from wild-type RbcL and mutants in proteobacteria and archaea by X-ray crystallography, Figure S1: Analysis Pipeline of RuBisCO using Bio3d package.
