# Supplementary material for: An Insight of RuBisCO Evolution through a Multilevel Approach
Source: Biomolecules. 2021 Nov 25;11(12):1761. doi: 10.3390/biom11121761 (PMC8698309; doi:10.3390/biom11121761)

biomolecules

MDPI

Supplementary data:

Table S1. 46 structures of isoforms I, II and III from wild-type RbcL and mutants in proteobacteria and archaea by X-ray crystallography.

RuBisCO form	Species	Lineage	PDB code	Organism	Resolution	No. Residues	% of structure resolved
IB	*Oryza sativa*	Higher plant	1wdd	Wild-tipe	1.35 Å	477	97
IB	*Triticum aestivum*	Higher plant	5wsk	Wild-tipe	1.78 Å	477	93
IB	*Pisum sativum*	Higher plant	4hhh	Wild-tipe	2.20 Å	475	96
IB	*Pisum sativum*	Higher plant	4mkv	Wild-tipe	2.15 Å	475	96
IB	*Spinacia oleracea*	Higher plant	1ir1	Wild-tipe	2.80 Å	475	97
IB	*Nicotiana tabacum*	Higher plant	4rub	Wild-tipe	2.70 Å	477	97
IB	*Nicotiana tabacum*	Higher plant	1rlc	Wild-tipe	2.70 Å	477	92
IB	*Nicotiana tabacum*	Higher plant	1rld	Wild-tipe	2.50 Å	477	92
IB	*Nicotiana tabacum*	Higher plant	1ej7	Wild-tipe	2.45 Å	477	95
IB	*Arabidopsis thaliana*	Higher plant	5iu0	Wild-tipe	1.50 Å	479	96
IB	*Chlamydomonas reinhardtii*	Green alga	1gk8	Wild-tipe	1.40 Å	475	98
IB	*Chlamydomonas reinhardtii*	Green alga	7jfo	Wild-tipe	2.13 Å	475	93
IB	*Chlamydomonas reinhardtii*	Green alga	2v6a	Mutant-Tipe	1.50 Å	475	98
IB	*Chlamydomonas reinhardtii*	Green alga	1uw9	Mutant-Tipe	2.05 Å	475	97
IB	*Chlamydomonas reinhardtii*	Green alga	2v68	Mutant-Tipe	2.30 Å	475	98
IB	*Chlamydomonas reinhardtii*	Green alga	2vdh	Mutant-Tipe	2.30 Å	475	97
IB	*Chlamydomonas reinhardtii*	Green alga	2vdi	Mutant-Tipe	2.65 Å	475	97
IB	*Chlamydomonas reinhardtii*	Green alga	2v69	Mutant-Tipe	2.80 Å	475	96
IB	*Synechococcus elongatus*	Cyanobacterium	6smh	Wild-tipe	4.30 Å	472	94
IB	*Nostoc sp*	Cyanobacterium	6kkm	Wild-tipe	3.00 Å	361	95
IB	*Thermosynechococcus elongatus BP-1*	Cyanobacterium	3zxw	Wild-tipe	2.10 Å	475	97
IB	*Synechococcus elongatus PCC 6301*	Cyanobacterium	1rsc	Wild-tipe	2.30 Å	472	98
IB	*Synechococcus elongatus PCC 6301*	Cyanobacterium	1rbl	Wild-tipe	2.20 Å	472	98
IC/D	*Skeletonema marinoi*	Diatoms	6ftl	Wild-tipe	2.60 Å	484	99
IC/D	*Chaetoceros socialis*	Diatoms	5oya	Wild-tipe	1.80 Å	490	95
IC/D	*Thalassiosira antarctica*	Diatoms	5mz2	Wild-tipe	1.90 Å	490	98
IC/D	*Galdieria partita*	Blue-green algae	1bwv	Mutant-Tipe	2.40 Å	493	95
IC/D	*Galdieria partita*	Blue-green algae	1iwa	Wild-tipe	2.60 Å	493	95
IA	*Cereibacter sphaeroides*	Proteobacterium	5nv3	Wild-tipe	3.39 Å	486	96
IA	*Halothiobacillus neapolitanus*	Proteobacterium	1svd	Wild-tipe	1.80 Å	473	94
IA	*Alcaligenes eutrophus*	Proteobacterium	1bxn	Wild-tipe	2.70 Å	486	92
IA	*Promineofilum breve*	Bacterium	6ura	Wild-tipe	2.17 Å	463	95
II	*Rhodopseudomonas palustris*	Proteobacterium	4lf1	Wild-tipe	2.38 Å	461	99
II	*Rhodopseudomonas palustris*	Proteobacterium	5han	Mutant-tipe	2.04 Å	461	99
II	*Rhodopseudomonas palustris*	Proteobacterium	5hjx	Mutant-tipe	1.80 Å	461	99
II	*Rhodopseudomonas palustris*	Proteobacterium	5koz	Mutant-tipe	2.30 Å	461	98
II	*Rhodopseudomonas palustris*	Proteobacterium	5hql	Mutant-tipe	2.53 Å	461	99
II	*Rhodopseudomonas palustris*	Proteobacterium	5hjy	Mutant-tipe	2.30 Å	461	98
II	*Rhodopseudomonas palustris*	Proteobacterium	5hat	Mutant-tipe	2.00 Å	461	98
II	*Rhodopseudomonas palustris*	Proteobacterium	5hao	Mutant-tipe	2.18 Å	461	98
II	*Rhodopseudomonas palustris, R. rubrum*	Proteobacterium	5hqm	Wild-tipe	1.95 Å	467	98
II	*Methanococcoides burtonii*	Archaea	5mac	Wild-tipe	2.60 Å	474	99
III	*Thermococcus kodakarensis*	Archaea	3a12	Wild-tipe	2.30 Å	444	98
III	*Thermococcus kodakarensis*	Archaea	3kdo	Mutant-tipe	2.36 Å	444	98

III	*Thermococcus kodakarensis*	Archaea	3wqp	Mutant-tipe	2.25 Å	444	98
III	*Thermococcus kodakarensis*	Archaea	3a13	Mutant-tipe	2.34 Å	444	98

Figure S1. Analysis Pipeline of RuBisCO using Bio3d packages. In this example the user starts with a protein identifier, performs a BLAST search to identify related structures, fetches and aligns the identified structures, performs PCA and calculates the ensemble normal mode analysis (eNMA). Then runs an MD for the selected proteins, and analyzes a PCA and DCCM to the simulation trajectories. The scripts, tutorials and result interpretation are available at http://thegrantlab.org/bio3d/ [21, 22].

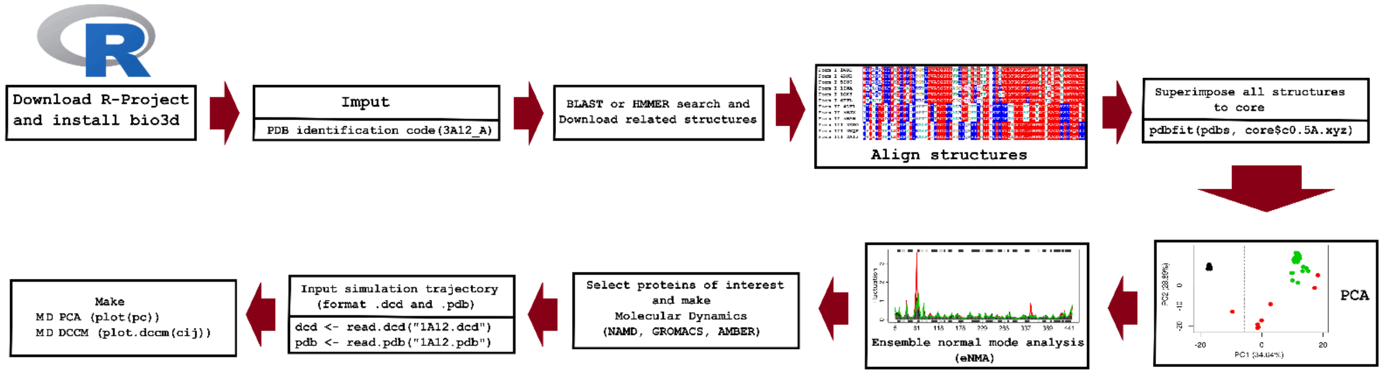

Supplement: Supplementary file 1 [file biomolecules-11-01761-s001.zip › biomolecules-1412441-supplementary.pdf]
